# Functional Differences of Glutamine Synthetase Isoenzymes in Wheat Canopy Ammonia Exchange

**DOI:** 10.3390/ijms27031179

**Published:** 2026-01-23

**Authors:** Xi Zhang, Junying Chen, Wenjing Song, Siddique Ahmad, Zhiyong Zhang, Huiqiang Li, Xinming Ma, Xiaochun Wang, Yihao Wei

**Affiliations:** 1 College of Life Science, Henan Agricultural University, Zhengzhou 450046, China; zhangxi010603@163.com (X.Z.); junyingchen6@163.com (J.C.); huiqiangli@henau.edu.cn (H.L.); 2 College of Agronomy, Henan Agricultural University, Zhengzhou 450046, China; wenjing15736795830@163.com (W.S.); siddiqueb284@gmail.com (S.A.); zhiyongzhang@henau.edu.cn (Z.Z.); maxinming@henau.edu.cn (X.M.); 3 Research and Experiment Station of Nitrogen and Phosphorus Loss in Farmland of the Yellow River Basin in Henan Province, Henan Agricultural University, Zhengzhou 450046, China

**Keywords:** ammonia compensation point, apoplastic NH_4_^+^, canopy ammonia exchange, glutamine synthetase, nitrogen metabolism, wheat

## Abstract

Canopy ammonia (NH_3_) exchange is a major contributor to agricultural NH_3_ emissions and is closely linked to nitrogen-use efficiency. Glutamine synthetase (GS) mediates plant NH_3_ assimilation, yet the specific roles of different GS isoenzymes in regulating wheat canopy NH_3_ exchange remain unclear. This study aimed to clarify the functional differences of wheat TaGS isoenzymes in modulating canopy–atmosphere NH_3_ exchange dynamics using two wheat cultivars (Yumai 49-198 and Xinong 509) under two nitrogen application levels (120 and 225 kg N ha^−1^). Field experiments combined with FTIR-based NH_3_ flux measurement, biochemical assays, and molecular analyses were conducted at anthesis and 16, 24, and 30 days after anthesis (DAA). Results showed that the leaf NH_3_ compensation point, determined by apoplastic NH_4_^+^ concentration, is a key factor influencing canopy NH_3_ exchange. Leaf NH_3_ sources exhibited distinct temporal specificity: photorespiration and nitrate reduction dominated at anthesis to 16 DAA, whereas nitrogenous compound degradation prevailed at 24–30 DAA. This temporal partitioning was highly coordinated with TaGS isoenzyme expression: TaGS2 was highest in early grain filling, potentially supporting assimilate NH_3_ from photorespiration/nitrate reduction, while TaGS1;1 expression increased progressively, aligning with the scavenging of NH_3_ from organic nitrogen degradation. These coordinated patterns suggest that the TaGS isoenzymes play differentiated roles in influencing wheat canopy NH_3_ exchange. This study thus provides correlative insights that point to potential molecular targets for breeding nitrogen-efficient wheat cultivars and mitigating agricultural NH_3_ emissions sustainably.

## 1. Introduction

Nitrogen is a fundamental macronutrient required for plant growth and development, and its application through fertilization remains a cornerstone of modern agriculture to ensure high and stable crop yields [1,2,3]. However, while intensified nitrogen use has significantly enhanced crop productivity, it has also resulted in considerable environmental challenges, with nitrogen losses from agricultural systems emerging as a major contributor to pollution [1,4,5]. These losses primarily occur through nitrate (NO_3_^−^) leaching (including surface runoff in rice systems), ammonia (NH_3_) volatilization, and nitrous oxide (N_2_O) emissions [6,7,8]. Each year, NH_3_ volatilization alone accounts for approximately 4.2–5.8 million tons of nitrogen loss (as elemental nitrogen), representing 15–20% of total agricultural nitrogen fertilizer applications [9]. Once in the atmosphere, NH_3_ can react with nitrogen oxides (NO_x_) and sulfur dioxide (SO_2_) to form ammonium nitrate (NH_4_NO_3_) and ammonium sulfate ((NH_4_)_2_SO_4_), key components of PM2.5 (Particulate Matter ≤ 2.5 μm), which contribute significantly to haze formation [10].

NH_3_ volatilization originates primarily from both the soil surface and crop canopies, with the latter functioning as a dynamic interface that can both emit and absorb atmospheric NH_3_ [11,12]. During the wheat growing season, canopy NH_3_ volatilization typically ranges from 0.8 to 1.5 kg ha^−1^, accounting for approximately 15–20% of total NH_3_ emissions from wheat fields [13]. In rice, canopy NH_3_ volatilization has been shown to be significantly and negatively correlated with nitrogen-use efficiency [14]. Studies further indicate that at the peak of leaf area development, corn canopies can absorb up to 76% of NH_3_ volatilized from the soil [15], while during the booting to anthesis stages in winter wheat, 23–40% of soil-emitted NH_3_ is absorbed by the canopy [16]. These findings highlight the critical role of canopy–atmosphere NH_3_ exchange in regulating nitrogen-use efficiency and mitigating agricultural NH_3_ losses.

The NH_3_ compensation point of crops is a critical factor regulating canopy–atmosphere NH_3_ exchange [17]. This compensation point refers to the atmospheric NH_3_ partial pressure at which there is no net flux of NH_3_ between the plant and the atmosphere, effectively corresponding to the intercellular NH_3_ partial pressure. When the plant’s NH_3_ compensation point is lower than the ambient atmospheric NH_3_ concentration, the plant absorbs NH_3_ from the air. Conversely, when the compensation point exceeds the surrounding NH_3_ concentration, the plant emits NH_3_ into the atmosphere.

Crop growth stage and nitrogen application rate have a significant impact on the NH_3_ compensation point. In winter wheat, the leaf NH_3_ compensation point peaks at approximately 60 nmol mol^−1^ during anthesis and the late grain-filling stages. Leaves from nitrogen-treated plants exhibit significantly higher NH_3_ compensation points than those from non-fertilized plants. At other growth stages, compensation points typically range from 1.3 to 16.0 nmol mol^−1^ [13]. Furthermore, studies have shown that barley cultivars with high glutamine synthetase (GS) activity have markedly lower leaf NH_3_ compensation points compared to cultivars with lower GS activity [18].

GS is a key enzyme responsible for NH_4_^+^ assimilation in plants, and is closely associated with crop canopy–atmosphere NH_3_ exchange. Extensive studies have shown that enhancing leaf GS activity facilitates cellular NH_4_^+^ assimilation, thereby lowering apoplastic NH_4_^+^ concentrations and reducing the leaf NH_3_ compensation point. This, in turn, promotes atmospheric NH_3_ uptake and mitigates canopy NH_3_ volatilization [16,19,20]. Furthermore, treatment with methionine sulfoximine (MSO), a GS-specific inhibitor, almost completely suppresses GS activity in plants [21].

In plants, GS is categorized based on subcellular localization into cytosolic GS (GS1) and plastidic GS (GS2) [22]. In wheat, *TaGS* genes comprise a family of 12 members, which are classified into four isoenzyme groups based on phylogenetic relationships: TaGS1;1, TaGS1;2, TaGS1;3, and TaGS2. Previous functional analyses of these isoenzymes have demonstrated notable differences in their enzymatic properties, tissue-specific localization, and expression patterns, highlighting their distinct roles in wheat nitrogen metabolism [23,24,25].

TaGS1;1 exhibits the highest affinity for NH_4_^+^ and is predominantly localized in leaf vascular tissues and mesophyll cells. Its expression markedly increases during leaf senescence, facilitating the re-assimilation of NH_4_^+^ released through protein degradation [23,24,25]. TaGS1;2, characterized by low NH_4_^+^ affinity but activation by its product glutamine, is primarily found in vascular bundles and plays a role in nitrogen re-translocation by assimilating NH_3_ derived from urea degradation [26]. Although TaGS1;3 shows relatively low expression in mesophyll cells, it demonstrates a high NH_4_^+^ assimilation capacity, with its expression induced by elevated NH_4_^+^ levels, suggesting a function in detoxifying excess NH_4_^+^ in mesophyll tissues [24,25]. TaGS2 is the dominant GS isoenzyme in leaves, localized in chloroplasts of mesophyll cells, where it re-assimilates NH_4_^+^ produced from photorespiration and assimilates NH_4_^+^ generated via NO_3_^−^ reduction [24,27]. Notably, TaGS2 expression declines sharply during leaf senescence, contributing significantly to the reduction in overall GS activity [28]. These findings underscore the distinct functional roles of the four TaGS isoenzymes in leaf nitrogen metabolism. However, their specific contributions to canopy–atmosphere ammonia exchange in wheat, and the identity of the isoenzyme most responsible for regulating this process, remain unresolved.

This study utilized previously identified wheat cultivars with contrasting nitrogen-use efficiencies in field experiments under varying nitrogen application levels [3,29]. Measurements included canopy–atmosphere ammonia exchange, leaf TaGS isoenzyme expression, nitrogen metabolic intermediate contents, and key enzyme activities. The relationships among TaGS isoenzyme expression patterns, nitrogen metabolism, and canopy NH_3_ exchange were analyzed across cultivars. The objective was to investigate the correlation between the four TaGS isoenzymes and canopy NH_3_ exchange in wheat. Findings aim to provide potential molecular targets for reducing agricultural NH_3_ volatilization, advancing green cultivation practices, and breeding nitrogen-efficient wheat varieties.

## 2. Results

### 2.1. Close Correlation Between Canopy Ammonia Exchange Dynamics and Leaf Ammonia Compensation Point

In-situ monitoring of wheat canopy–atmosphere NH_3_ exchange was conducted using the GT5000 portable multi-parameter soil respiration measurement system. The net canopy NH_3_ flux was determined by a sequential measurement approach: the total NH_3_ flux from both the wheat plants and the soil was first measured. Subsequently, the aboveground wheat biomass was carefully removed, and the soil NH_3_ flux was measured again at the same location. The difference between the two measurements was calculated to represent the net NH_3_ flux attributable specifically to the wheat canopy, with positive values indicating net emission and negative values indicating net uptake.

**Figure 1:**
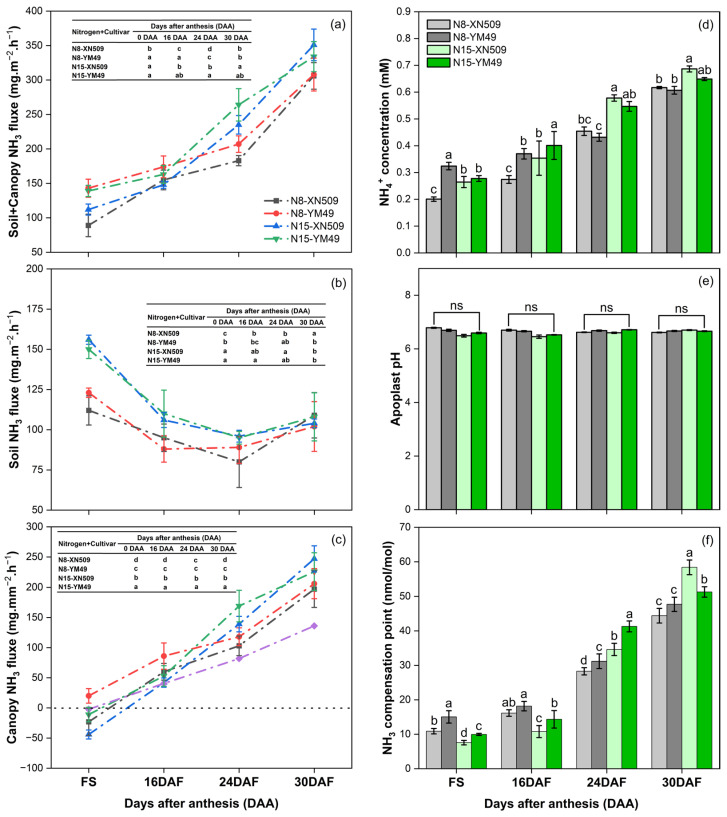
Different letters indicate significant differences at the 5% probability level according to the Tukey test. Ammonia (NH_3_) fluxes from the plant-soil system: (**a**) soil + canopy, (**b**) soil only, and (**c**) wheat plants alone, as influenced by different wheat varieties, growth stages, and nitrogen treatments. Panels (**d**,**e**) show corresponding NH_4_^+^ concentrations and apoplast pH levels, while (**f**) presents NH_3_ compensation points across varieties, developmental stages, and nitrogen application rates. Abbreviation: AS = Anthesis stage. Data are the mean of three replicates.

This method revealed distinct temporal dynamics and treatment-specific patterns in canopy NH_3_ flux during the grain-filling period (Figure 1). In addition, no condensation was generated on the walls of the sampling chamber during the sampling process. Canopy NH_3_ flux was lowest at the anthesis stage and progressively increased with plant development. Most treatments exhibited NH_3_ absorption at the anthesis stage, with the N15-treated XN509 cultivar showing the highest absorption. At 24 and 30 DAA, the N15 treatment resulted in significantly greater canopy NH_3_ volatilization compared to the N8 treatment (Figure 1c).

Cultivar-specific differences were stage-dependent: at 16 and 24 DAA, YM49 showed higher canopy NH_3_ volatilization than XN509, whereas at 30 DAA under the N15 treatment, XN509 exhibited significantly greater volatilization than YM49. Canopy NH_3_ flux (Figure 1c) was closely correlated with the leaf NH_3_ compensation point (Figure 1f). When atmospheric NH_3_ concentrations fell below the leaf NH_3_ compensation point, the canopy released NH_3_; conversely, when atmospheric levels exceeded this threshold, the canopy absorbed NH_3_. To elucidate the physiological mechanisms underlying these differences, the leaf ammonia compensation points and their influencing factors were further analyzed. Apoplastic pH values remained consistent across treatments (6.4–6.7), showing no significant variation (Figure 1e), while apoplastic NH_4_^+^ content steadily increased with developmental progression (Figure 1d). The leaf NH_3_ compensation point followed a similar trend, being lowest at anthesis and increasing gradually during development. Notably, after 24 days post-anthesis, the N15 treatment exhibited significantly higher NH_3_ compensation points than N8. Cultivar comparisons revealed that from anthesis to 24 DAA, XN509 had a lower NH_3_ compensation point than YM49. However, at 30 DAA under the N15 treatment, YM49 displayed a significantly lower compensation point than XN509 (Figure 1f). These findings highlight apoplastic NH_4_^+^ content as a key factor influencing the leaf NH_3_ compensation point.

### 2.2. Photorespiration and Nitrate Reduction as Primary Sources of Leaf Ammonia During Early Grain Filling

**Figure 2:**
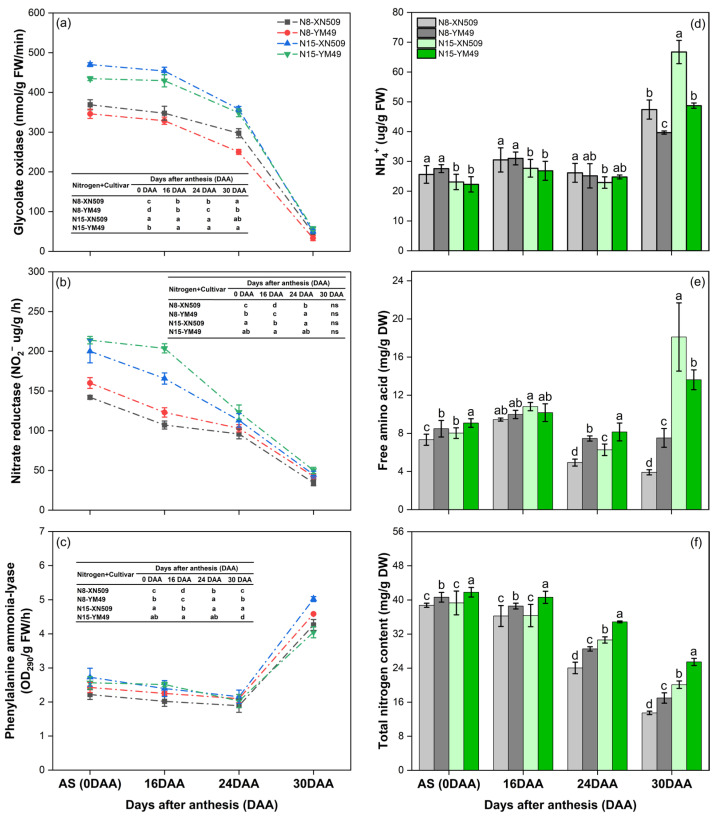
Data are the mean of three replicates. Different letters indicate significant differences at the 5% probability level according to the Tukey test. Enzymatic activities and nitrogen metabolites in wheat varieties across growth stages under different nitrogen treatments: (**a**) Glycolate oxidase activity (nmol g^−1^ FW min^−1^), (**b**) Nitrate reductase activity (μg NO_2_^−^ g^−1^ FW h^−1^), and (**c**) Phenylalanine ammonia-lyase activity (OD_290_ g^−1^ FW h^−1^). Panels (**d**–**f**) show changes in nitrogen metabolites, including (**d**) ammonium (NH_4_^+^), (**e**) free amino acids, and (**f**) total nitrogen content across wheat varieties, stages, and nitrogen levels. Abbreviations: AS = Anthesis stage; FW = Fresh weight; DW = Dry weight.

The apoplastic NH_4_^+^ content is regulated by leaf nitrogen metabolism, with a particular focus on the balance between NH_4_^+^ production and assimilation. Glycolate oxidase (GO), a key enzyme marker of photorespiratory metabolism, consistently exhibited high activity during early grain filling, especially in the XN509 cultivar (Figure 2a). At the same time, nitrate reductase (NR) activity, which catalyzes the reduction of nitrate (NO_3_^−^) to nitrite (NO_2_^−^) as the first step in inorganic nitrogen assimilation, also showed high activity, particularly under the N15 treatment and in the YM49 cultivar (Figure 2b). This elevated NR activity indicates an enhanced flux through the nitrate reduction pathway, ultimately contributing to the production of free NH_4_^+^. In contrast, phenylalanine ammonia-lyase activity remained low, suggesting limited ammonia release via phenylalanine degradation during this phase (Figure 2c). These findings highlight the significant role of photorespiration and nitrate reduction pathways in the production of free NH_4_^+^ in leaves during early grain filling.

The efficient coupling of NH_4_^+^ production with assimilation and subsequent translocation during early grain filling is indicated by the low and comparable levels of free NH_4_^+^ and amino acid measured at AS and 16 DAA (Figure 2d,e). The low levels of both free NH_4_^+^ and amino acids imply rapid assimilation of photorespiratory and nitrate-derived NH_4_^+^ into amino acids, coupled with their efficient export from the leaf. The maintenance of relatively constant total leaf nitrogen content during early grain filling further supports this interpretation (Figure 2f), indicating that nitrogen-containing compounds synthesized in the flag leaf are actively translocated to developing grains rather than accumulating locally.

Nitrogen application level significantly influenced nitrogen metabolism, with the N15 treatment sustaining higher NR activity and thus greater nitrate reduction capacity (Figure 2b). Cultivar comparisons revealed distinct metabolic profiles: XN509 exhibited higher photorespiratory capacity, as indicated by GO activity, while YM49 showed enhanced NR activity (Figure 2a). These differences reflect cultivar-specific pathways of NH_3_ production during early grain filling.

### 2.3. Degradation of Nitrogen-Containing Organic Compounds Becomes the Primary Source of Leaf Ammonia During Late Grain Filling

During late grain filling (24 to 30 DAA), the sources of leaf NH_3_ shifted markedly. GO activity declined precipitously, with activity at 30 DAA dropping to approximately 20% of that observed at 24 DAA (Figure 2a), signaling a substantial reduction in photorespiration-derived NH_3_ production. Concurrently, NR activity exhibited a gradual but consistent decrease throughout this developmental phase (Figure 2b), further diminishing the contribution of nitrate reduction to leaf NH_3_ generation. In stark contrast, phenylalanine ammonia-lyase activity increased dramatically at 30 DAA, with the N15-treated XN509 cultivar exhibiting the highest activity among all treatments (Figure 2c). This indicates that phenylalanine catabolism was activated and likely became a significant source of NH_3_.

At 30 DAA, both free NH_4_^+^ and free amino acid contents increased markedly, especially in N15-treated XN509 (Figure 2d,e). This coincided with a rapid decline in total leaf nitrogen content (Figure 2f), indicating extensive degradation of leaf proteins and other nitrogen-containing compounds into amino acids, some of which were further catabolized to release NH_4_^+^. The produced NH_4_^+^ exceeded the leaf’s assimilation capacity, leading to accumulation of free NH_4_^+^. These findings suggested that degradation of nitrogen-containing organic compounds becomes the primary source of leaf ammonia during late grain filling.

**Figure 3:**
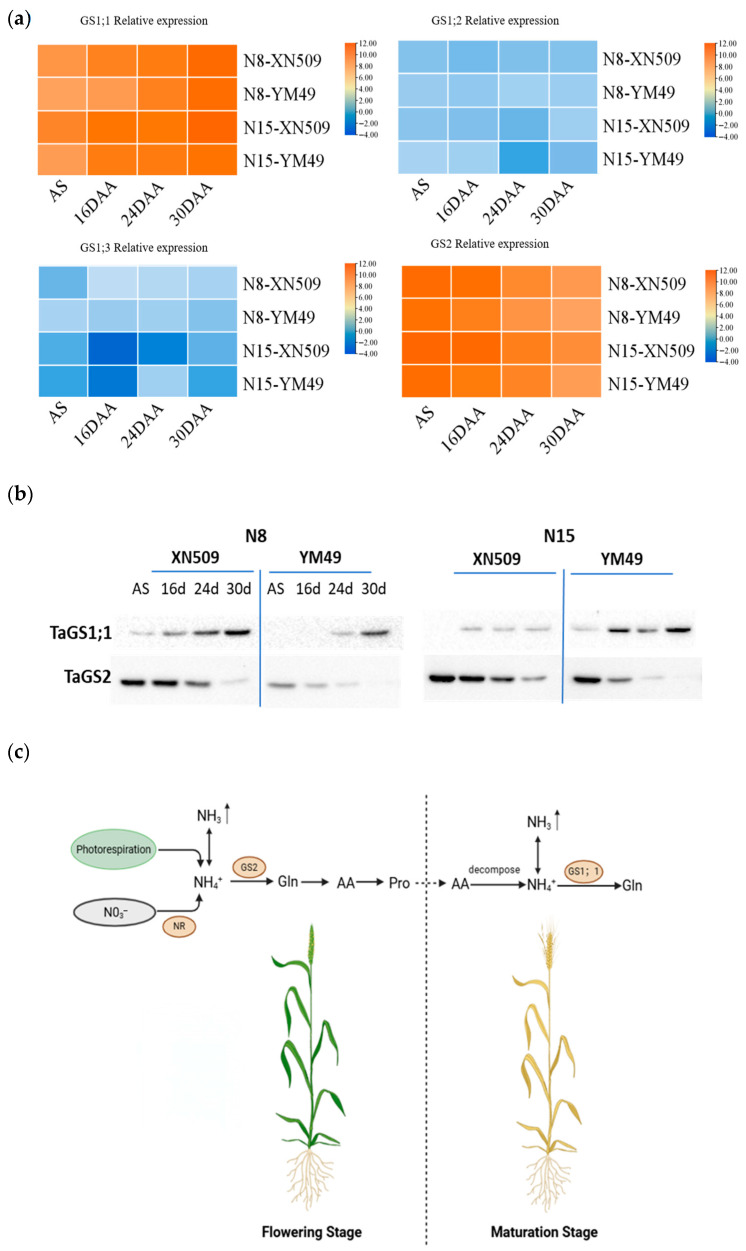
Expression analysis of TaGS isoenzymes in different cultivars under varying nitrogen treatments across developmental stages. (**a**) qPCR analysis of TaGS isoenzyme gene expression. The heatmap was created to visualize the expression profiles of TaGS genes across different developmental stages, considering both cultivar variation and nitrogen (N) fertilization, based on qRT-PCR data. The geometric mean of Ct values for TaATPases and TaTEF was used to normalize the expression ratios of each gene. Data represent the means of three independent biological replicates. Gene expression data are presented as log2-transformed values of the normalized data.; (**b**) Western blot analysis of TaGS1;1 and TaGS2 proteins. (**c**) Diagram of the functional pattern of glutamine synthetase isoenzyme in canopy ammonia exchange in wheat. Unidirectional arrows: substance conversion; bidirectional arrows: mutual transformation; NH_3_↑: ammonia volatilization; dashed lines: separation of two growth stages.

Nitrogen application level and cultivar characteristics exerted complex effects during late grain filling. High nitrogen supply (N15) delayed the decline of leaf nitrogen metabolism but simultaneously accelerated degradation of nitrogen-containing organic compounds (Figure 2f), significantly increasing apoplastic NH_4_^+^ content and leaf NH_3_ compensation points (Figure 1d,f), thereby promoting canopy NH_3_ volatilization (Figure 1c). Cultivar differences were pronounced, with XN509 exhibiting more extensive degradation of nitrogen compounds under high nitrogen conditions (Figure 2f), resulting in free NH_4_^+^ accumulation (Figure 3a), elevated apoplastic NH_4_^+^ levels (Figure 1d), and greater NH_3_ volatilization (Figure 1c). This explains why canopy NH_3_ volatilization in XN509 was significantly higher than in YM49 at 30 DAA under N15 treatment.

### 2.4. Spatiotemporal Differences in TaGS Isoenzyme Expression and Coordination with Ammonia Sources

Glutamine synthetase (GS), the key enzyme for NH_3_ assimilation, plays a critical role in regulating canopy NH_3_ exchange. This study found that total leaf GS activity gradually declined as development progressed, with significant differences among treatments. Throughout grain filling, XN509 exhibited higher GS activity than YM49, and activity under N15 treatment exceeded that under N8.

Analysis of the four TaGS isoenzymes revealed that leaves predominantly expressed *TaGS1;1* and *TaGS2*, while *TaGS1;2* and *TaGS1;3* were expressed at very low levels (Figure 3a). *TaGS1;1* and *TaGS2* displayed distinct stage-specific and complementary expression patterns: *TaGS2* expression steadily decreased over time, with the highest level at anthesis and reaching its lowest at 30 DAA; conversely, *TaGS1;1* expression increased progressively, reaching a maximum at 30 DAA. This complementary pattern was confirmed at both transcriptional and protein levels (Figure 3b), indicating these isoenzymes fulfill different physiological roles at various developmental stages.

TaGS isoenzyme expression showed clear temporal coordination with NH_3_ sources. During early grain filling, when leaf NH_3_ mainly originated from photorespiration and nitrate reduction, TaGS2 expression was highest. Since TaGS2 localizes in chloroplasts, where photorespiration and nitrate reduction produce NH_4_^+^, higher TaGS2 expression facilitates efficient NH_4_^+^ assimilation from these pathways. This maintains lower apoplastic NH_4_^+^ content and leaf NH_3_ compensation point, reducing NH_3_ volatilization or even promoting NH_3_ absorption (Figure 3c).

In contrast, during late grain filling, NH_3_ is predominantly derived from amino acid and other organic nitrogen degradation occurring mainly in the cytosolic, coinciding with a significant increase in TaGS1;1 expression. TaGS1;1 localizes primarily in the cytosolic, enabling effective assimilation of NH_4_^+^ released from organic nitrogen catabolism. Elevated TaGS1;1 expression helped mitigate the rise in apoplastic NH_4_^+^ content during late grain filling, thus controlling leaf NH_3_ compensation point and reducing NH_3_ volatilization (Figure 3c).

Nitrogen application level significantly influenced TaGS isoenzyme expression, with N15 treatment generally enhancing both TaGS1;1 and TaGS2 levels. Cultivar comparison showed that XN509 consistently exhibited higher TaGS isoenzyme expression than YM49, aligning with its higher total GS activity and explaining why XN509 had a lower leaf NH_3_ compensation point and greater NH_3_ absorption capacity during early grain filling.

Integrating these findings, wheat canopy NH_3_ exchange is directly regulated by the leaf NH_3_ compensation point, which depends on apoplastic NH_4_^+^ content. Apoplastic NH_4_^+^ content reflects the balance between leaf NH_3_ production and assimilation. Throughout grain filling, the sources of leaf NH_3_ and the assimilation mechanisms undergo marked shifts. During early grain filling, NH_3_ derived primarily from photorespiration and nitrate reduction was temporally coupled with the high expression of TaGS2, suggesting a major role for this isoenzyme in its assimilation. Conversely, in late grain filling, NH_3_ from organic nitrogen degradation coincided with the progressively increasing expression of TaGS1;1, implying its predominant role during this phase (Figure 3c). This spatiotemporally coordinated metabolic regulation is vital for improving nitrogen-use efficiency and reducing NH_3_ volatilization losses.

## 3. Discussion

### 3.1. Leaf Ammonia Compensation Point as the Key Factor Influencing Wheat Canopy Ammonia Exchange

When atmospheric NH_3_ concentration is below the leaf NH_3_ compensation point, the canopy releases NH_3_; conversely, when atmospheric NH_3_ concentration exceeds the compensation point, the canopy absorbs NH_3_ [30,31]. Based on analyses across developmental stages, nitrogen treatments, and cultivars, this study confirms that the leaf NH_3_ compensation point is the primary factor determining both the direction and magnitude of canopy NH_3_ exchange.

Given that the apoplastic pH remained within a stable and narrow range (6.4–6.7) across all treatments, its direct influence on modulating the NH_3_ compensation point appears to have been limited under these specific conditions. Consequently, the observed variations in the leaf NH_3_ compensation point were likely driven predominantly by changes in the apoplastic NH_4_^+^ content, which showed a close correspondence with the compensation point trends. These findings align with those of Mattsson and Schjoerring, who reported that variations in apoplastic NH_4_^+^ concentration directly explain differences in plant NH_3_ volatilization under different nitrogen regimes [16]. Changes in apoplastic NH_4_^+^ concentration reflect the balance between NH_3_ production and assimilation within leaf cells, processes jointly regulated by plant developmental stage, nitrogen nutritional status, and genotype [32].

Importantly, the wheat leaf NH_3_ compensation point increased significantly with developmental progression, consistent with a shift in canopy NH_3_ exchange from net absorption to net release. This dynamic change highlights a substantial transformation in plant NH_3_ metabolic regulation across developmental stages and is crucial for understanding nitrogen metabolism and transport during crop growth.

### 3.2. Stage-Specific Transformation of Wheat Leaf Ammonia Sources

This study revealed dynamic shifts in wheat leaf NH_3_ sources during grain filling. During early grain filling, leaf NH_3_ primarily originated from photorespiration and nitrate reduction, whereas during late grain filling, it mainly derived from the degradation of amino acids, proteins, and other nitrogen-containing organic compounds. These transformations closely reflect changes in plant physiological functions.

In early grain filling, GO and NR activities were high, indicating active photosynthetic and nitrogen metabolic processes. Photorespiration is a major NH_3_ source in C_3_ plant leaves, with its inhibition strongly suppressing nitrate assimilation [33]. NR-catalyzed nitrate reduction also releases substantial NH_3_, especially under high nitrogen supply [34]. NH_3_ produced by these processes is primarily localized within chloroplasts or organelles closely associated with chloroplasts.

During late grain filling, GO and NR activities declined significantly, while phenylalanine ammonia-lyase activity increased sharply, coinciding with substantial rises in leaf free NH_4_^+^ and amino acid contents. This indicates that as leaves senesce, protein and nitrogen-containing macromolecule degradation become the main NH_3_ sources. Up to 90% of proteins in senescing leaves are degraded, and most of the nitrogen derived from this degradation is transported to growing organs via xylem and phloem. [35]. NH_3_ produced during this phase is primarily localized in the cytoplasm, contrasting with earlier chloroplast localization.

Nitrogen supply significantly influenced the dynamics of leaf NH_3_ sources. High nitrogen treatment (N15) sustained greater NR activity and photorespiratory capacity, while also promoting the accumulation and subsequent degradation of nitrogen-containing organic compounds during late grain filling. These findings align with Xu, who reported nitrogen supply effects on plant nitrogen metabolic enzymes and nitrogen redistribution [34]. Cultivar differences were also evident in NH_3_ production pathways, likely related to variations in photosynthetic efficiency, nitrogen uptake and utilization efficiency, and nitrogen transport capacity [36].

### 3.3. Spatiotemporal Coordination Between TaGS Isoenzyme Expression and Ammonia Sources as the Molecular Basis for Maintaining Nitrogen Metabolic Balance

A core finding of this study is the significant spatiotemporal coordination between wheat TaGS expression and leaf NH_3_ sources. During early grain filling, TaGS2 expression was highest. Since TaGS2 is primarily localized in chloroplasts, it spatially coincides with NH_3_ produced from photorespiration and nitrate reduction. Conversely, during late grain filling, TaGS1;1 expression increased significantly. TaGS1;1, predominantly localized in the cytoplasm, spatially corresponds to NH_3_ released from the degradation of nitrogen-containing organic compounds.

This precise spatiotemporal coordination reflects sophisticated plant regulatory mechanisms for assimilating NH_3_ from distinct sources. GS2 is the principal enzyme that assimilates NH_3_ produced via photorespiration and nitrate reduction within chloroplasts [37]. GS2-deficient mutants exhibit severe growth inhibition and accumulation of photorespiratory intermediates under normal atmospheric conditions. In contrast, multiple GS1 isoenzymes show diverse spatiotemporal expression and functions in plants [22]. Our findings that TaGS1;1 expression significantly increases during leaf senescence align with the observed role of its homolog in maize [38], suggesting that TaGS1;1 primarily assimilates NH_3_ released from protein degradation during senescence.

Notably, although TaGS1;1 expression increases during late grain filling, it remains insufficient to fully assimilate NH_3_ generated from extensive organic nitrogen degradation, leading to free NH_4_^+^ accumulation and elevated apoplastic NH_4_^+^ content. This imbalance likely underlies the increased canopy NH_3_ volatilization observed during late grain filling. The imbalance between NH_3_ production and assimilation in leaves is a key determinant of NH_3_ volatilization intensity [32]. This imbalance is especially pronounced in senescing leaves due to extensive protein degradation coupled with declining overall GS activity [39].

Wheat cultivars exhibited significant differences in TaGS isoenzyme expression patterns. Cultivar XN509 showed generally higher TaGS isoenzyme expression, particularly TaGS2 during early grain filling and TaGS1;1 during late grain filling, compared to YM49. These differences likely stem from genetic background and nitrogen utilization strategy [40]. Previous research indicates that high nitrogen-use efficiency cultivars typically possess enhanced nitrogen assimilation capacity and more coordinated nitrogen metabolic regulation mechanisms [41]. These genotypic variations offer important molecular targets for wheat cultivar improvement.

### 3.4. Relationships Between TaGS Isoenzyme Expression and Canopy Ammonia Exchange and Agricultural Application Significance

This study confirmed a close relationship between TaGS isoenzyme expression and canopy NH_3_ exchange. Higher TaGS isoenzyme expression levels facilitate assimilation of leaf-produced NH_3_, maintaining lower apoplastic NH_4_^+^ content and leaf ammonia compensation point, thereby reducing leaf NH_3_ volatilization or promoting NH_3_ absorption. These findings align with previous studies; for example, reported that barley cultivars with high GS activity exhibited significantly lower leaf NH_3_ compensation points compared to cultivars with low GS activity [42]. Similarly, demonstrated that GS1-overexpressing transgenic rice showed reduced ammonia compensation points and decreased NH_3_ volatilization [19].

However, this study also revealed complexities in the relationship between TaGS isoenzyme expression and canopy NH_3_ exchange. During late grain filling, even XN509, which exhibited higher TaGS isoenzyme expression, showed significant NH_3_ volatilization under high nitrogen conditions. This indicates that when NH_3_ production exceeds assimilation capacity, elevated TaGS expression alone cannot fully suppress NH_3_ volatilization. This observation is consistent with previous research emphasizing the decisive role of nitrogen metabolic balance in regulating NH_3_ volatilization [30].

These results carry important implications for agricultural practices. First, cultivating and selecting wheat cultivars with high TaGS isoenzyme expression and strong nitrogen assimilation capacity may lower leaf ammonia compensation points, reduce canopy NH_3_ volatilization, and enhance nitrogen-use efficiency. Thomsen highlighted that improving crop GS expression through marker-assisted selection or genetic engineering is an effective strategy for boosting nitrogen-use efficiency [22].

Second, optimizing nitrogen fertilizer management is critical to minimizing wheat canopy NH_3_ volatilization. While high nitrogen supply (N15) enhanced TaGS isoenzyme expression, it simultaneously promoted extensive degradation of nitrogen-containing organic compounds during late grain filling, ultimately increasing NH_3_ volatilization. This underscores the importance of controlling nitrogen inputs and avoiding excessive late-stage nitrogen applications.

Finally, the findings suggest that balancing NH_3_ production and assimilation in wheat is essential for effective regulation of canopy NH_3_ exchange. Beyond enhancing TaGS isoenzyme expression, strategies such as moderating protein and nitrogen-containing macromolecule degradation during late grain filling or promoting efficient nitrogen transport may further reduce NH_3_ volatilization losses. These approaches offer promising directions for future wheat cultivar improvement and cultivation management optimization.

Several methodological limitations should be considered in this study. First, the use of a static acrylic chamber for in-situ NH_3_ flux measurement may lead to a slight underestimation of flux due to NH_3_ adsorption onto the chamber walls. Second, the canopy flux separation procedure involving plant removal may temporarily alter the local microenvironment, introducing uncertainty into flux calculations. Additionally, the NH_3_ compensation points reported here are model-based estimates derived from apoplastic NH_4_^+^ and pH. Their calculation relies on specific assumptions and may be influenced by dynamic field conditions, which carry inherent uncertainty. Despite these potential limitations, the consistent and significant treatment responses observed in both flux and biochemical data strongly support the robustness of the key comparative conclusions drawn regarding cultivar differences and nitrogen management effects.

## 4. Materials and Methods

### 4.1. Experimental Design

Field experiments were conducted at the Xuchang Science and Education Park, Henan Agricultural University, using a split-plot design with two factors: nitrogen levels and wheat cultivars. The basic soil fertility was as follows: organic matter 2.53%, total nitrogen 1.2 mg/g, total phosphorus 0.7 mg/g, total potassium 18.6 mg/g, available phosphorus 1.1 mg/kg, and available potassium 192 mg/kg. The main plots comprised two nitrogen levels: 120 kg N ha^−1^ (N8) and 225 kg N ha^−1^ (N15). Nitrogen was supplied in the form of urea (46% N), corresponding to urea application rates of 260.87 kg ha^−1^ for the N8 treatment and 489.13 kg ha^−1^ for the N15 treatment. In addition, a basal fertilizer dressing was applied uniformly across all plots, consisting of 857.14 kg ha^−1^ of superphosphate (14% P_2_O_5_) and 200 kg ha^−1^ of potassium chloride (60% K_2_O). Subplots included two wheat cultivars: Yumai 49-198 (YM49) and Xinong 509 (XN509). Each plot measured 7.5 m × 12 m, and treatments were replicated three times. Sowing was done on October 15 at a seeding rate of 150 kg ha^−1^ with 20 cm row spacing. All other agronomic practices followed standard high-yield management protocols.

### 4.2. Sampling and Monitoring Methods

Sampling and measurements were performed at anthesis, as well as at 16, 24, and 30 days after anthesis (DAA). Climate information during the wheat growing season is presented in Supplementary Figure S1. No rainfall was recorded at the sampling time points of the anthesis, as well as at 16, 24, and 30 days after anthesis (DAA), and the corresponding climate details are listed in Supplementary Table S1. To account for meteorological conditions during the wheat growth period, representative rainless days were carefully selected for sampling and monitoring. The measurements were performed daily between 10:00 a.m. and 5:00 p.m. Ammonia exchange between the canopy and the atmosphere, along with soil ammonia volatilization, were measured using a Gasmet GT5000 Terra Fourier Transform Infrared (FTIR) GHG analyzer (Gasmet Technologies Oy, Vantaa, Finland) and a clear, cubic acrylic chamber (0.5 m × 0.5 m × 0.5 m) within a closed system, with a 1 Hz sampling rate (Figure 1). The GT5000 Terra can simultaneously detect multiple gases (NH_3_, H_2_O, CO, CO_2_, N_2_O) by scanning the full infrared spectrum and calculating the concentration of each gas based on its absorption, with a precision of ±3%. FTIR technology allows for the identification of distinct peaks and regions within the measurement spectrum, reducing the risk of cross-interference between gases. Zero-point calibration with nitrogen (N_2_) was conducted immediately before and after each measurement to minimize any background signals or measurement offsets. The base of the chamber is a 0.6 m × 0.6 m metal frame, which was buried 10 cm into the soil three days prior to ammonia measurements. The chamber was ventilated before each measurement and placed on top of the base, then sealed using a water seal. Following chamber placement, measurements were taken over a 5-min period to capture a stable change in gas concentration. During the measurement process, an external gas cooling device was used to maintain the temperature inside the chamber in alignment with the ambient temperature.

**Figure 4:**
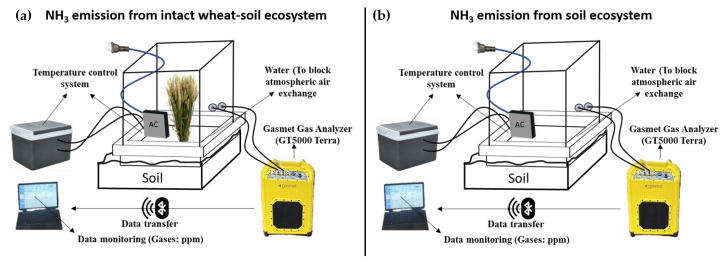
Schematic illustration of the plant cutting method and data acquisition process used to measure NH_3_ emissions. Panel (**a**) shows the intact wheat plant-soil system, while panel (**b**) depicts the setup immediately after plant cutting to isolate soil-emitted NH_3_. Emissions were recorded using the Gasmet GGA G5000 Terra analyzer, with real-time data displayed and transferred to a connected portable device for analysis.

In each plot, three monitoring points were randomly selected along the seeding rows. Initially, total ammonia flux was measured with the chamber placed over intact wheat plants (Figure 4a). Subsequently, the aboveground wheat biomass within the chamber area was removed, and soil ammonia volatilization was measured under the same conditions (Figure 4b). Canopy ammonia flux was then calculated by subtracting soil ammonia volatilization from the total ammonia flux recorded with plant coverage.

Aboveground wheat parts removed from the chamber coverage areas were collected, and functional or flag leaves were selected for analysis. Intact fresh leaves were used to determine apoplastic NH_4_^+^ concentration and apoplastic pH. The leaf NH_3_ compensation point was calculated based on these measurements and corresponding meteorological data. Remaining leaf samples were immediately flash-frozen in liquid nitrogen and stored at −80 °C for subsequent analyses. These included the expression profiling of TaGS isoenzymes, nitrate reductase (NR), phenylalanine NH_3_ lyase, and glycolate oxidase (GO); quantification of total nitrogen, NH_4_^+^, NO_3_^−^, and free amino acid contents; and measurement of GS activity.

### 4.3. Leaf Apoplastic Solution Extraction

Rinse fresh leaves with deionized water and blot them dry with filter paper. Weigh approximately 5 g of the leaves, place them in a 200 mL syringe, block the water outlet, and add 150 mL of 280 mmol/L sorbitol solution. Make most parts of the leaves turn dark green through processes such as aspiration, liquid discharge, and squeezing. Remove the leaves, blot the surface liquid dry, then centrifuge at 1000× *g* for 10 min at 4 °C to obtain the apoplastic extract. Determine the pH value of the apoplastic solution using a pH meter, and measure the NH_4_^+^ concentration by the indophenol blue colorimetry method [43].

### 4.4. Wheat Leaf Ammonia Compensation Point Calculation

When K_d_≪[H^+ ]_apo (apoplastic H^+^ concentration), the calculation formula for leaf NH_3_ compensation point at 25 °C is:

(1) $$X_{s}=\alpha \times K_{H}\times K_{d}$$

where X_s_ is leaf NH_3_ compensation point; α is the ratio of apoplast NH_4_^+^ concentration to apoplastic H^+^ concentration; K_H_ and K_d_ are thermodynamic constants, 10^−1·76^ L mol^−1^ and 10^−9·25^ L mol^−1^, respectively, at 25 °C.

Leaf NH_3_ compensation point at actual temperature was calculated using:

(2) $$\ln\frac{X_{T}}{X_{s}}=\frac{\left(∆H_{dis}^{0}+∆H_{vap}^{0}\right)}{R}\times \left(\frac{1}{T_{ref}}-\frac{1}{T}\right)$$

where X_T_ is leaf NH_3_ compensation point at actual temperature; X_s_ is leaf NH_3_ compensation point at 25 °C; ΔH^0^_dis_ is NH_4_^+^ dissociation enthalpy (52.21 kJ mol^−1^); ΔH^0^_vap_ is vaporization enthalpy (34.18 kJ mol^−1^); R is gas constant (0.00831 kJ K^−1^ mol^−1^); T_ref_ is 298.15 K (25 °C); T is actual temperature (K) during field sampling [44].

### 4.5. Free Ammonium Nitrogen Content Determination

Place approximately 0.2 g of leaf samples ground with liquid nitrogen into a 2 mL centrifuge tube, add 1 mL of 1% acetic acid, vortex to mix thoroughly, shake for extraction at 4 °C for 15 min, and then centrifuge at 13,000× *g* at 4 °C for 15 min. Pipette 0.8 mL of the supernatant into a 10 mL centrifuge tube, then add 7 mL of distilled water, 1 mL of color developing reagent, 200 μL of sodium hypochlorite solution, and 200 μL of 10 g/L sodium nitroprusside solution in sequence, mix well, and react at room temperature for 1 h. For the blank control, replace 0.8 mL of the supernatant with 0.8 mL of distilled water in the above reaction system. Pipette 0.20 mL of the reaction solution into a 96-well microplate, set up at least 3 replicates for each sample, and measure the absorbance value at 697 nm using a microplate reader.

### 4.6. Nitrate Nitrogen Content Determination

Place approximately 0.2 g of leaf samples ground with liquid nitrogen into a 5 mL centrifuge tube, add 2 mL of 10% trichloroacetic acid (TCA), vortex to mix thoroughly, shake for extraction at room temperature for 20 min, and then centrifuge at 13,000× *g* for 15 min. Pipette 0.5 mL of the supernatant into a 10 mL centrifuge tube, add 0.3 mL of salicylic acid solution, mix well, and react at room temperature for 20 min. Next, add 7 mL of 2 mol/L NaOH, mix thoroughly, and cool to room temperature. For the blank control, replace 0.5 mL of the supernatant with 0.5 mL of 10% TCA in the above operation process. Pipette 0.20 mL of the reaction solution into a 96-well microplate, set up at least 3 replicates for each sample, and measure the absorbance value at 410 nm using a microplate reader.

### 4.7. Free Amino Acid Content Determination

Place approximately 0.2 g of leaf samples ground with liquid nitrogen into a 2 mL centrifuge tube, add 1 mL of 10% acetic acid, vortex to mix thoroughly, shake for extraction on ice for 10 min, and then centrifuge at 13,000× *g* at 4 °C for 10 min. Pipette 0.15 mL of the supernatant into a 10 mL centrifuge tube. Next, prepare the reaction system by mixing acetic acid buffer solution, ninhydrin solution, and 0.4% ascorbic acid solution at a ratio of 2:3:0.1; after mixing well, pipette 5.1 mL of this system into the 10 mL centrifuge tube, heat it in a boiling water bath for 15 min, and then cool it to room temperature. For the blank control, replace 0.15 mL of the supernatant with 0.15 mL of distilled water in the above reaction solution. Pipette 0.20 mL of the reaction solution into a 96-well microplate, set up at least 3 replicates for each sample, and measure the absorbance value at 570 nm using a microplate reader.

### 4.8. Total Nitrogen Content Determination

Weigh 0.1 g of the dried sample (accurate to 0.0001 g), add 5 mL of sulfuric acid with a concentration of 1.84 g/mL, and let it stand overnight. The next day, digest the sample in a digestion furnace at 380 °C until it turns dark brown. Add 10 drops of hydrogen peroxide, and after digesting for 20 min, add another 7–8 drops of hydrogen peroxide. Observe the color of the sample in the digestion tube until it becomes clear. After the final addition of hydrogen peroxide, continue digesting for another 30 min. Allow the digested sample to cool to room temperature, dilute it to a constant volume of 50 mL with distilled water, and then let it stand for 10 min. Pipette 1 mL of the supernatant, mix it with 3 mL of distilled water in a 5 mL centrifuge tube, and then analyze the mixture using an AA3 flow analyzer (SEAL, Norderstedt, Germany) [28].

### 4.9. Determination of Total GS Activity in Wheat Leaf

Take approximately 0.5 g of fresh tissue samples ground into powder with liquid nitrogen, add 1.5 mL of extraction buffer (100 mM Tris-HCl, 1 mM EDTA, 1 mM MgCl_2_, 10 mM β-mercaptoethanol, and 1 mM PMSF, pH 7.6), vortex to mix thoroughly, and extract at low temperature for 20 min. After centrifuging at 13,000× *g* and 4 °C for 30 min, collect the supernatant, which is the crude enzyme solution. Transfer 200 µL of the crude enzyme solution into a 5 mL centrifuge tube, and add the following components of the reaction system in sequence: 0.6 mL of imidazole-HCl buffer (0.25 M, pH 7.0), 0.4 mL of sodium glutamate (0.3 M, pH 7.0), 0.4 mL of ATP-Na_2_ (30 mM, pH 7.0), 0.2 mL of MgSO_4_ (0.5 M), and 0.2 mL of Tris-HCl (0.1 M, pH 7.6). After mixing well, incubate in a water bath at 25 °C for 5 min. Add 0.2 mL of hydroxylamine (prepared by mixing 1 M hydroxylamine hydrochloride and 1 M NaOH in equal volumes), and incubate in a water bath at 25 °C for 15 min. Finally, add 0.8 mL of FeCl_3_ reagent, mix thoroughly to terminate the reaction. Then, centrifuge the reaction mixture at 13,000× *g* for 5 min at room temperature, and measure the absorbance at a wavelength of 540 nm.

### 4.10. Nitrate Reductase, Phenylalanine Ammonia-Lyase, and Glycolate Oxidase Activity Determination

NR activity was determined using the same crude enzyme extract as used for glutamine synthetase (GS) activity, with NR activity measured via the sulfanilamide colorimetric method.

For phenylalanine ammonia-lyase (PAL) activity, PAL catalyzes the conversion of L-phenylalanine to trans-cinnamic acid. The latter has a characteristic absorption peak at 290 nm, and the activity of PAL can be determined by the production of trans-cinnamic acid. A total of 0.2 g of liquid nitrogen-ground sample was extracted with 1 mL of 5 mmol L^−1^ mercaptoethanol borate buffer, shaken, and centrifuged at 4 °C, 9500× *g* for 15 min. The supernatant served as a crude enzyme extract. Two 5 mL centrifuge tubes were prepared: the test contained 0.5 mL extract, 1 mL 20 mmol L^−1^ phenylalanine, and 2 mL distilled water; the control used 1 mL of 50 mmol L^−1^ borate buffer (pH 8.8) instead of phenylalanine. Both were incubated at 30 °C in the dark for 30 min. After adding 0.1 mL of 5 mol L^−1^ HCl to stop the reaction, tubes were centrifuged at room temperature (9500× *g*, 5 min), and absorbance at 290 nm was recorded. PAL activity was expressed as absorbance change per hour at 290 nm [45].

GO activity was measured following the instructions of a commercial kit (Suzhou Grace Biotechnology Co., Ltd., Suzhou, China), based on the absorbance of glyoxylate phenylhydrazone at 324 nm. GO activity was expressed as enzyme units catalyzing the formation of 1 nmol glyoxylate phenylhydrazone per gram tissue per minute.

### 4.11. TaGS Gene Expression Analysis

Total RNA was extracted from plant tissue using HiPure HP Plant RNA Kit B (Guangzhou Magen Biotechnology Co., Ltd., Guangzhou, China). cDNA was synthesized using the RTIII Super Mix with dsDNase (Monad Biotech Co., Ltd., Shanghai, China). Quantitative real-time PCR (qPCR) was performed on a Step One Real-Time PCR System (Life Technologies Corporation, Carlsbad, CA, USA) with SYBR Green qPCR Mix (Monad) for the assay. All primers (Sangon Biotech Co., Ltd., Shanghai, China) used are shown in Supplementary Table S2. The qPCR mix was composed of 10 µL SYBR Green qPCR Mix (Monad), 5 µL diluted cDNA 1:10 (*v*/*v*), 0.5 µL and 10 µM forward and reverse primers, respectively, and 4 µL of sterile nuclease-free water. Reactions proceeded according to the following program: 95 °C for 10 min, followed by 40 cycles of 95 °C for 15 s, 58 °C for 15 s, and 72 °C for 20 s. Fluorescence readings were taken during the elongation step (72 °C). Melting curves were obtained from 60 to 95 °C with a 0.5 °C increase every 15 s. Relative expression levels of genes were calculated using *TaATPases* (*Ta54227*) and *TaTEF* (*Ta53964*) genes as internal control [46].

### 4.12. TaGS Isoenzyme Western Blot Analysis

Western blot analysis of TaGS was performed as previously described [24]. Approximately 0.3 g of the fine homogeneous powder was mixed with 0.9ml of Extraction buffer (100 mM Tris, 1 mM EDTA, 1 mM MgCl2, 1 mM phenylmethanesulfonyl fluoride (PMSF), and 10 mM β-mercaptoethanol; pH 7.6) by shaking at 4 °C for 10 min. The extract was centrifuged at 12,000× *g* at 4 °C for 30 min. The supernatant was then prepared for further experiments.

A total of 5 μg of soluble proteins extracted from the grain were loaded onto each lane. Proteins were separated in 12.5% (*w*/*v*) polyacrylamide gel and electrophoretically transferred to a 0.45 μm pore-size PVDF membrane (Merck Millipore Ltd., Darmstadt, Germany) in transfer buffer (25 mM Tris-base and 192 mM Gly, 10% methanol) at 200 mA for 50 min. The membranes were blocked with TBST (20 mM Tris-base, 150 mM NaCl, and 0.05% (*v*/*v*) Tween 20, pH 7.4) containing 5% skimmed milk at 4 °C overnight. The membrane was incubated at 20 °C for 1.5 h with the TaGS1;1, TaGS1;2, TaGS1;3, and TaGS2 antibodies, respectively, and the dilution ratios of antibody is 1:10,000, 1:30,000, 1:30,000, 1:10,000, and 1:50,000, respectively. In previous studies, the specificity of four TaGS isoenzyme antibodies has been validated. After three times washing with TBST, the membrane was incubated at room temperature for 1 h with horseradish peroxidase-conjugated goat anti-rabbit IgG (ABclonal Biotechnology Co., Ltd., Wuhan, China) at 1:25,000. After several washes with TBST, the membrane was incubated at room temperature for 5 min using Clarity Western ECL reagent (Bio-Rad, Hercules, CA, USA), and the signals were detected by ChemiDocTM XRS+ Imaging System (Bio-Rad).

## 5. Conclusions

This study revealed that the expression of TaGS isoenzymes (TaGS2 and TaGS1;1) showed a temporal correlation with canopy NH_3_ exchange dynamics in wheat. Specifically, a distinct shift was observed: NH_3_ sources in early grain filling, dominated by photorespiration and nitrate reduction, were correlated with high TaGS2 expression. Conversely, NH_3_ from organic nitrogen degradation in late grain filling was correlated with increased TaGS1;1 expression. These associations suggest that TaGS2 and TaGS1;1 may have distinct, stage-preferential roles in NH_3_ assimilation, highlighting potential molecular targets for sustainable wheat breeding and emission mitigation.

## Data Availability

The data supporting the findings of this study are included in the Supplementary Materials. Additional details are available from the corresponding author upon request.

## Supplementary Materials

The following supporting information can be downloaded at: https://www.mdpi.com/article/10.3390/ijms27031179/s1.

## Abbreviations

The following abbreviations are used in this manuscript:

NH_3_	ammonia
NUE	nitrogen-use efficiency
GS	glutamine synthetase
DAA	days after anthesis
AS	Anthesis stage
N	nitrogen
NH_4_^+^	ammonium
FTIR	Fourier Transform Infrared Spectroscopy
PM2.5	Particulate Matter ≤ 2.5 μm
MSO	methionine sulfoximine
NR	nitrate reductase
GO	Glycolate oxidase
PAL	Phenylalanine ammonia-lyase
