# Supplementary material for: Functional Differences of Glutamine Synthetase Isoenzymes in Wheat Canopy Ammonia Exchange
Source: Int J Mol Sci. 2026 Jan 23;27(3):1179. doi: 10.3390/ijms27031179 (PMC12897959; doi:10.3390/ijms27031179)

Table S1. Temperature and precipitation at the anthesis, as well as at 16, 24, and 30 days after anthesis

**Title 1**	Mean rainfall(mm	Mean temperture(℃)
2024/3/20	0	19.47
2024/24	0	21.88
2024/5/10	0	24.76
2024/5/30	0	28.26

Table S2. List of primers used for qPCR.

Gene Name	Primer	Sequence(5’-3’)
TaGS1;1	*TaGS1;1-F*	AAGGACGGCGGGTTC AA
	TaGS1;1-R	GCGATGTGCTCCTTGTGCTT
TaGS1;2	*TaGS1;2-F*	GACAACTTCCTTGTTATGTGCCAC
	TaGS1;2-R	TGTGCCTCTTGTTCGTGGG
TaGS1;3	*TaGS1;3-F*	CTGTGACTGCTATGCGCCTAAC
	TaGS1;3-R	CCGCGTTGTACCGCTTGT
TaGS2	*TaGS2-F*	GGTTGACAGGGCTACACGAGA
	TaGS2-R	GAGCAGCCACGGTTCGC
ATPase	*ATPase-S*	ATACGCCATCAGGGAGAACATC
	ATPase-A	AGGGTTGTCCTTCCTCCGC
TaEF1	*TaEF1-S*	GGTTGTGGAGACCTTTGCTACTTAC
	TaEF1-A	AACAGCCACAGTTTGCCTCAT

Figure S1. Climatic factors during the wheat growing season

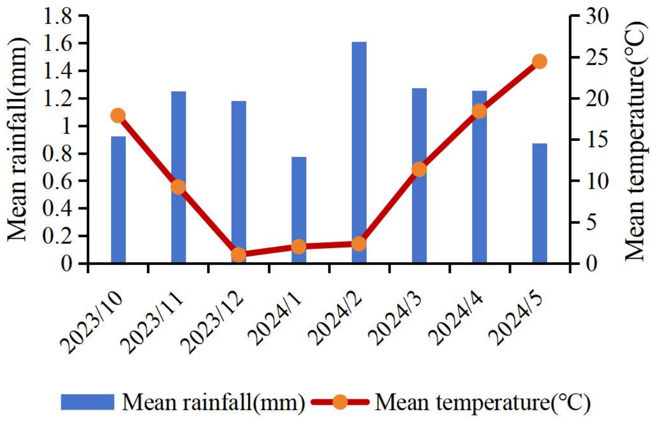

Supplement: Supplementary file 1 [file ijms-27-01179-s001.zip › ijms-4081677-supplementary.pdf]
